# New version of the Epidemic Intelligence Information System for food- and waterborne diseases and zoonoses (EPIS-FWD) launched

**DOI:** 10.2807/1560-7917.ES.2016.21.49.30422

**Published:** 2016-12-08

**Authors:** Celine M Gossner

**Affiliations:** 1 European Centre for Disease Prevention and Control (ECDC), Stockholm, Sweden

On 1 December 2016 the third version of the Epidemic Intelligence Information System for food- and waterborne diseases and zoonoses (EPIS-FWD) was launched. With this development, the European Centre for Disease Prevention and Control (ECDC) moved one step further towards the One Health approach.

In collaboration with the European Food Safety Authority (EFSA), the Molecular Typing Cluster Investigation (MTCI) module was expanded to also allow the assessment of Salmonella, Shiga toxin-producing *Escherichia coli* (STEC) and *Listeria monocytogenes* microbiological clusters based on non-human isolates (i.e. food, feed, animal and environmental) and on a mix of non-human and human isolates.

Depending on the type of cluster assessed, the MTCIs are coordinated by ECDC or EFSA or jointly by both agencies together with public health and/or food safety and veterinary experts from the involved European Union (EU) and European Economic Area (EEA) Member States.

ECDC collects human typing data through the European Surveillance System (TESSy) since 2013 [1]. Typing data from non-human isolates can now be submitted by the food and veterinary authorities of the EU/EEA Member States through the EFSA molecular typing data collection system. Furthermore, the joint ECDC-EFSA molecular typing database allows the comparison of the typing data collected by ECDC and EFSA.

First launched in March 2010, the Epidemic Intelligence Information System for food- and waterborne diseases and zoonoses (EPIS-FWD) has become an important tool for assessing on-going public health risks related to FWD events worldwide. Currently, 52 countries from five continents have access to the outbreak alerts in the EPIS-FWD [2].

Since its launch, 305 outbreak alerts have been assessed through the EPIS-FWD; 32 (10%) were from countries outside of the EU/EEA which underlines the global dimension of the system.

The Health Security Committee, a part of the European Commission and the officially nominated public health risk management authority in the EU/EEA, has access to the EPIS-FWD to ensure the link between risk assessment and risk management. The World Health Organisation (WHO), including the International Network of Food Safety Authorities (INFOSAN) managed jointly by the Food and Agriculture Organisation of the United Nations (FAO) and WHO, is invited to contribute to the discussions in the EPIS-FWD when international outbreaks involve non-EU/EEA countries.

Through this new version of EPIS-FWD, ECDC and EFSA are encouraging the sharing of data between sectors and aspire to strengthen the multi-sectorial collaboration at international and national levels.
